# Emergence of melioidosis in Brazil: a case series

**DOI:** 10.1186/s13256-023-04093-8

**Published:** 2023-08-23

**Authors:** Dilbert Silva Velôso, Samuel Pinheiro da Silva, Conceição Maria Sousa de Coelho, José Miguel Luz Parente, Thaline Alves Elias Veloso, Murilo Moura Lima, Caroline Torres Sampaio, Maria Fátima Alencar Bezerra de Freitas, Dionne Bezerra Rolim, Elba Regina Sampaio de Lemos, Marco Aurélio Pereira Horta

**Affiliations:** 1 University Hospital/Federal University of Piaui, Teresina, Brazil; 2 Federal University of Ceará, Fortaleza, Brazil; 3 Laboratório de Hantaviroses e Rickettsioses/Instituto Oswaldo Cruz/Fundação Oswaldo Cruz, Rio de Janeiro, Brazil

**Keywords:** *Burkholderia pseudomallei*, Melioidosis, Brazil

## Abstract

**Background:**

Melioidosis is a serious disease caused by the bacterium *Burkholderia pseudomallei* which affects humans and animals. It results in a wide spectrum of clinical manifestations, mainly in the respiratory tract, progressing to septic shock and death.

**Case presentation:**

Herein, we present a series of seven patients (median age: 41 years) with confirmed melioidosis, treated at a public hospital in Piauí State, Brazil between 2019 and 2021. The most common clinical presentations were fever, cough, pneumonia, and abdominal pain. The mean duration of antibacterial therapy with 1 g of meropenem was 28.6 ± 1.1 days. Six patients recovered and one died. The mean hospitalization time was 51.0 ± 39.2 days.

**Conclusions:**

Melioidosis is an emerging infectious disease in Brazil. Health professionals in endemic areas need to be aware of the clinical presentation and appropriate clinical management of the disease because early diagnosis and early initiation of antibiotic therapy can be life-saving.

## Background

Melioidosis is an infectious and neglected disease caused by the bacteria *Burkholderia pseudomallei*, a Gram-negative bacillus that naturally inhabits soils and water that occurs predominantly in tropical regions [1–3]. It can infect humans or animals and is mainly transmitted through skin contact, mucous membranes, inhalation, or ingestion [4]. Vertical transmission, maternal milk transmission and sexual transmissions have also been reported [5, 6]. In Brazil, the disease was first recorded in 2003 in an outbreak in Ceará State, where three of the four patients with a severe form of the disease died [7]. Awareness regarding melioidosis is still low in Brazil, making the disease underreported [7]. Patients infected with *B. pseudomallei* may develop severe pneumonia and sepsis. Diabetes mellitus (DM), chronic lung and renal diseases are known risk factors [6] for occurrence of fatal cases of melioidosis if not promptly diagnosed and treated. In the absence of rapid intensive treatment, patients can develop severe pulmonary involvement, and urinary and skin infections, arthritis, osteomyelitis, and, less frequently, neuromelioidosis. Bacteremia occurs in 40–60% of cases, sometimes without an evident focus of infection, and septic shock with pneumonia occurs in 20% of cases [8]. We report the first case series of seven laboratory-confirmed cases of melioiosis in Piauí State, Brazil.

## Case presentations

The cases are summarized in Table 1 and described below.

**Table 1:** Clinical and laboratorial findings of cases of meliodosis in Piauí State, Brazil

Case no.	Age/sex	Clinical presentation	Clinical features	BP isolated from	(HB) g/dL	(HT) %	LEUC × 10^3^/µL	PLAT × 10^3^/µL	URE mg/dL	CRE mg/dL	CRP mg/dL	Na + mmol/L	K + mmol/L	Outcome
1	50/F	MS, pneumonia	Dyspnea, productive cough, without fever	Urine	12.1	38.2	7.88	296	45.6	0.25	4.13	130.0	4.7	Recovered
2	80/M	ITP	Petechial rash, abdominal pain, and dyspnea	Blood	6.5	21.1	24.0	5	110	1.13	160.9	133.0	3.7	Death
3	22/M	DM type 1, SLE, fibromyalgia, osteomyelitis	Abdominal pain, leg weakness, fever	Blood	8.0	25.0	5.43	458	52.1	1.08	105.26	137.0	4.6	Recovered
4	63/F	Hypertension, CKD, history of liver transplantation, tuberculosis	Weakness, productive cough, fever	Blood	6.9	20.3	3.7	107	126.6	3.35	137.91	130.0	5.1	Recovered
5	38/F	DM type 1, hypothyroidism	Productive cough, daily afternoon fever, abdominal pain, weight loss	BALF	8.6	25.8	11.52	392	48	0.91	138	3.9	17.25	Recovered
6	25/M	DM type 1, pyelonephritis	Fever, asthenia, pain in the right hypochondrium	Liver abscess	7.5	22.9	9.02	450	22.3	0.63	146.83	123.0	4.0	Recovered
7	41/M	DM type 2	Fever, chills, pale feces, dark urine, dry cough, leg edema	Blood, liver abscess	10.9	32.7	12.45	198	27.0	1.03	184.79	128	4.1	Recovered

All patients were self-identified as “pardos”, of visibly mixed racial origin

*BALF* bronchoalveolar lavage fluid, *BP* *Burkholderia pseudomallei*, *CKD* chronic kidney disease, *CRE* creatinine, *DM* diabetes mellitus, *HB* hemoglobin, *HT* hematocrit, *ITP* idiopathic thrombocytopenic purpura, *LEUC* leukocytes, *MS* multiple sclerosis, *PLAT* platelets, *SLE* systemic lupus erythematosus, *URE* urea

### Case 1

A 50-year-old woman with a history of multiple sclerosis (MS) was admitted to hospital in February 2019 with pneumonia that progressed to respiratory failure. The patient was underwent tracheostomy, gastrostomy, and antibiotic therapy with piperacillin/tazobactam was started after collection of blood and urine samples for culture. The urine sample was positive for *B. pseudomallei* on culture. The antibiotic regiment was changed to ceftazidime and meropenem. She was discharged from the hospital in July 2019 after 139 days with feeding via gastrostomy and passage of urine via a urinary catheter; next to application of aforementioned antibiotics, the patient was treated with risperidone, lamotrigine, scopolamine, and omeprazole.

### Case 2

An 80-year-old man with hypertension was admitted to hospital in February 2019 with a 3-month history of a petechial rash on the arms and abdomen, associated with pruritus. The patient was diagnosed with steroid refractory idiopathic thrombocytopenic purpura (ITP). His condition did not improve, and 2 weeks after admission he underwent a chest computed tomography (CT), which showed lesions suggestive of alveolar hemorrhage, requiring positive pressure ventilation and mechanical compression of the pulmonary vessels to reduce alveolar bleeding. Six weeks after admission, the patient developed fever (38.5 °C), abdominal pain, and dyspnea. Blood culture was positive for *B. pseudomallei* and the patient was treated with meropenem. However, he developed airway hemorrhage and cardiorespiratory arrest, resulting in the patient’s death the following day, 43 days after admission.

### Case 3

A 22-year-old man with a history of systemic lupus erythematosus (SLE) and fibromyalgia was hospitalized on March 2019 due to abdominal pain and weakness in the legs. Magnetic resonance imaging revealed the presence of free fluid in the abdominal cavity and a pericardial effusion. Two weeks after admission, antibiotic therapy with meropenem was started and subsequently changed to sulfamethoxazole/trimethoprim (SMZ/TMP) after a positive blood culture for *B. pseudomallei*. The patient developed fever, intense pain in the right iliac fossa, and the right sacroiliac joint, which restricted walking. Pelvic magnetic resonance imaging showed an inflammation suggestive of osteomyelitis. Laborataroy analysis showed proteinuria (764.2 mg/24 h), and anti-nuclear antigen (ANA) positive (titer: 1:1280) and anti-DNA antibodies (titer: 1:40). The patient was diagnosed with class IV lupus nephritis. His condition deteriorated, with persistent fever (39.5 °C) associated with hypotension, and a decreased level of consciousness. He was admitted to the ICU and after 44 days of hospitalization, he was discharged and continued treatment with SMZ/TMP, hydroxychloroquine, and prednisone.

### Case 4

A 63-year-old woman was admitted to hospital in October 2019 due to weakness, productive cough, and fever (38 °C), for 2 weeks. She was hypertensive, diabetic and had chronic renal disease with history of liver transplantation due to alcoholic cirrhosis and treatment for tuberculosis. The patient received a Blood transfusion and antibiotic therapy with ceftriaxone was started, but the patient developed acute respiratory failure. Chest CT showed pleural effusion on the right, compressive atelectasis of the adjacent alveolar parenchyma associated with ground-glass opacities in several lung fields. Blood culture was positive for *B. pseudomallei.* The patient was treated with meropenem and was discharged one month later.

### Case 5

A 38-year-old woman with a history of DM was admitted to hospital in December 2020 with hypothyroidism, cough, fever, abdominal pain, and weight loss. Chest CT showed excavated lesions in the left lung with extensive homogeneous parenchymal opacities in the lower lobe and in the posterior segment of the upper lobe of the right lung. Bronchoscopy was performed, and the culture of the bronchoalveolar lavage fluid was positive for *B. pseudomallei* with sensitivity to SMZ/TMP, ceftazidime, and meropenem. The patient was discharged 33 days after admission and prescribed SMZ/TMP for a further 3 months.

### Case 6

A 25-year-old diabetic patient was admitted to hospital in July 2021 with fever, asthenia, and pain in the right hypochondrium and right lumbar region that radiated to the right inguinal region, for 25 days. Reported pyelonephritis improved after cefepime and vancomycin. CT of the abdomen and chest showed three expansive lesions in the liver, compatible with liver abscesses and bilateral pleural effusion with compressive atelectasis of the underlying lung parenchyma. On 08/23/21, the patient underwent drainage of abscesses, and the culture of the sample was positive for *B. pseudomallei*. After treatment with meropenem the patient was discharged after 32 days of hospitalization.

### Case 7

**Fig. 1:**
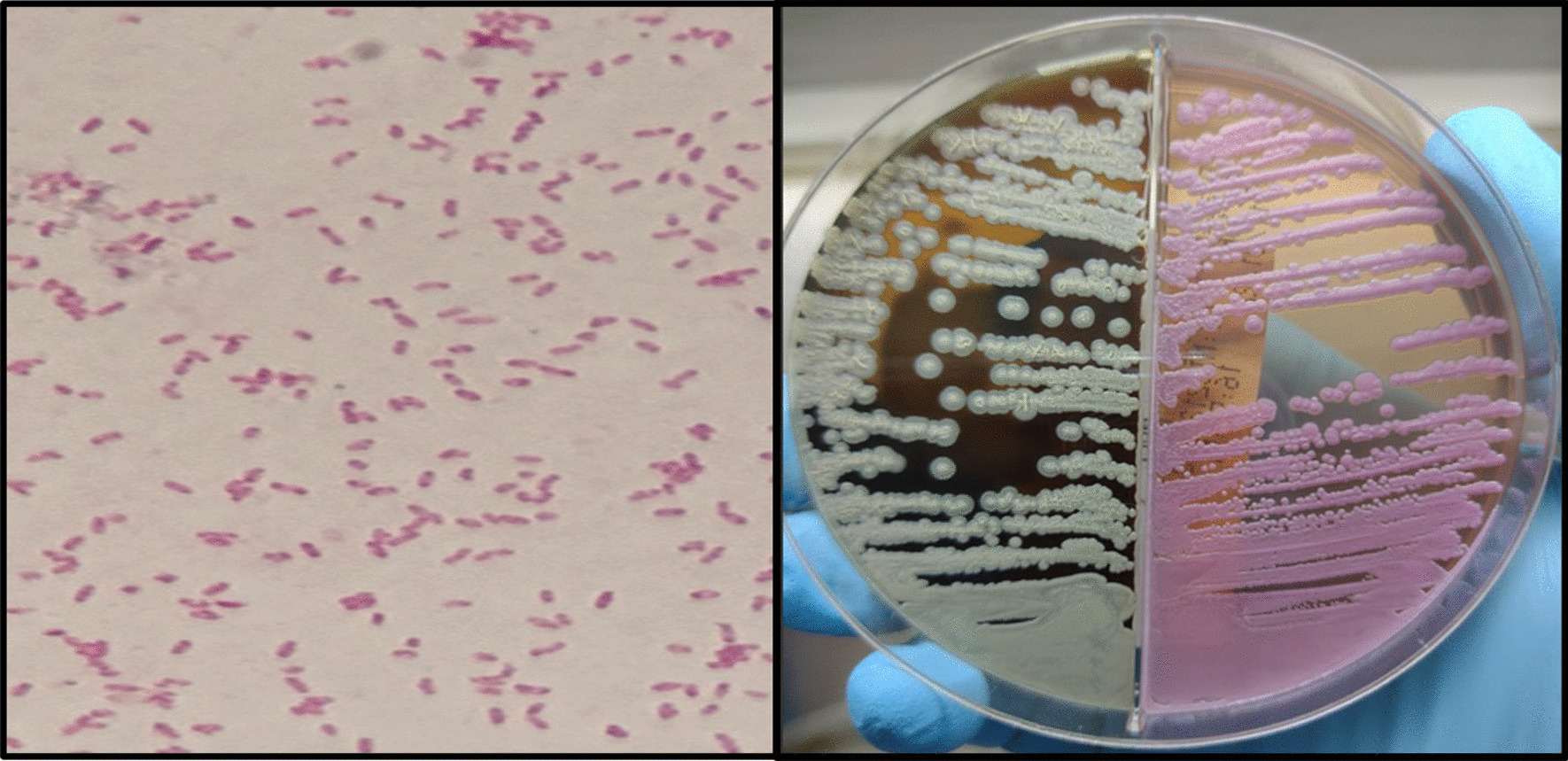
Left: Gram-negative bacilli of *Burkholderia pseudomallei* with characteristic hyperchromatic bipolar staining. Right: *Burkholderia pseudomallei* isolates on blood agar and MacConkey agar from liver abscess contents

A 41-year-old man with a history of type 1 DM was admitted to hospital in October 2021 with fever (39 °C) for more than 1 month, chills, dry cough, pale feces, and dark urine. The patient had edema of the legs and chest CT showed a hypodense heterogeneous lesion in the right hepatic lobe, segments VIII and V, and splenomegaly. Empiric antibiotic therapy was started with ciprofloxacin and metronidazole. Blood culture was positive for *B. pseudomallei* and the antibiotic was switched to meropenem (Fig. 1). Abdominal CT confirmed the presence of free fluid in the upper abdomen and a bilateral pleural effusion. Culture of pus from the liver abscess was also positive for *B. pseudomallei* and this was confirmed by molecular analysis. The patient was discharged from hospital after 29 days.

## Discussion

This is the first documented case series of patients diagnosed with melioidosis in Piauí State, Brazil. Three patients were females and four were males, with ages ranging from 22 to 80 years. All patients had comorbidities: DM (4 patients), SLE (1 patient), MS (1 patient), and ITP (1 patient). They presented different manifestations including pulmonary involvement, abscesses, and skin involvement, with a mortality rate of 14% (1/7). Other studies have reported fatality rates of 5.7%, 20.5%, and 32.2% [10, 11]. In addition to alcoholism [9], and DM [10], the most common risk factors associated with melioidosis are: (i) advanced age, (ii) exposure to soil and (iii) water contaminated with *B. pseudomalle*, mainly workers who carry out agricultural activities, and (iv) comorbidities such as chronic kidney and lung diseases, heart disease, and thalassemia [10]. We observed that the cases were not related to each other and the possible infection source of infection could not be determined in the study. In relation to clinical manifestations, the patients had in common: fever (6), cough (4), abdominal pain (4), dyspnea (2) and leg weakness (2).The results also showed a similar pattern, with most patients having a pre-existing metabolic disorder, which may have contributed to the clinical development of melidioidosis. Other case reports are presented in Table 2. Regarding the diagnosis, the bacterium was isolated from blood (Cases 2, 3, 4, and 7), urine (Case 1), bronchioalveolar lavage fluid (Case 5), and liver abscesses (Cases 6 and 7), and all samples were seeded in culture media specific for clinical specimens. After growth, the isolates were subjected to automated identification, however only four isolates were sent for molecular testing (Cases 4, 5, 6, and 7), most likely because of a lack of prior knowledge of melioidosis in this region, and the lack of established flows and protocols for the referral of patients with infectious diseases.

**Table 2:** Distribution of melioidosis cases reported between 2021 and 2022, according to melioidosis.info [12]

Year	Location	Source			Deaths
		Environment	Human	Animal	
2021	Australia (553), Bangladesh (2), Brunei (1), Colombia (16), Congo (3), India (93), Laos (16), Madagascar (1), Malaysia (10), Mali (1), Myanmar (11), Nepal (2), Nicaragua (1), Philippines (1), Singapore (15), Sri Lanka (1), Thailand (292)	304	715	–	95
2022	Australia (398), Bangladesh (1), Benin (1), China (1), Ghana (56), India (737), Malaysia (47), Panama (1), Philippines (2), Sri Lanka (4), Thailand (2574), United States of America (4), Viet Nam (3)	574	3254	1	787

## Conclusions

Melioidosis is an emerging and neglected disease in Brazil and its diagnosis needs to be investigated in patients with risk factors, such as DM and renal failure. It needs to be considered in the differential diagnosis of other diseases with a similar clinical picture, such as abscesses caused by *M. tuberculosis*, certain *Salmonella enterica* spp, and *Treponema pallidum*. Given the high prevalence of tuberculosis in Brazil, increased surveillance is necessary because these two diseases require different antibiotic treatment. This first cases series of melioidosis to be reported in Piauí State highlights the need to develop a structured network with a trained team and an adequate structure of the laboratories for the investigation and detection of *B. pseudomallei* infection, which has a high case fatality rate in the absence of timely and specific treatment.

## Data Availability

All data generated or analysed during this study are included in this published article.

## Abbreviations

CT: Computed tomography
DM: Diabetes mellitis
ICU: Intensive care unit
ITP: Idiopathic throbocytopenic purpura
MS: Multiple sclerosis
SLE: Systemic lupus erythematosis
SMZ/TMP: Sulfamethoxazole/trimethoprim
